# Supplementary material for: Spatiotemporal biocontrol and rhizosphere microbiome analysis of Fusarium wilt of banana
Source: Commun Biol. 2023 Jan 11;6:27. doi: 10.1038/s42003-023-04417-w (PMC9834294; doi:10.1038/s42003-023-04417-w)
Supplement: Supplementary file 2 — Supplementary Information [file 42003_2023_4417_MOESM2_ESM.pdf]

Supplementary Figures and Tables

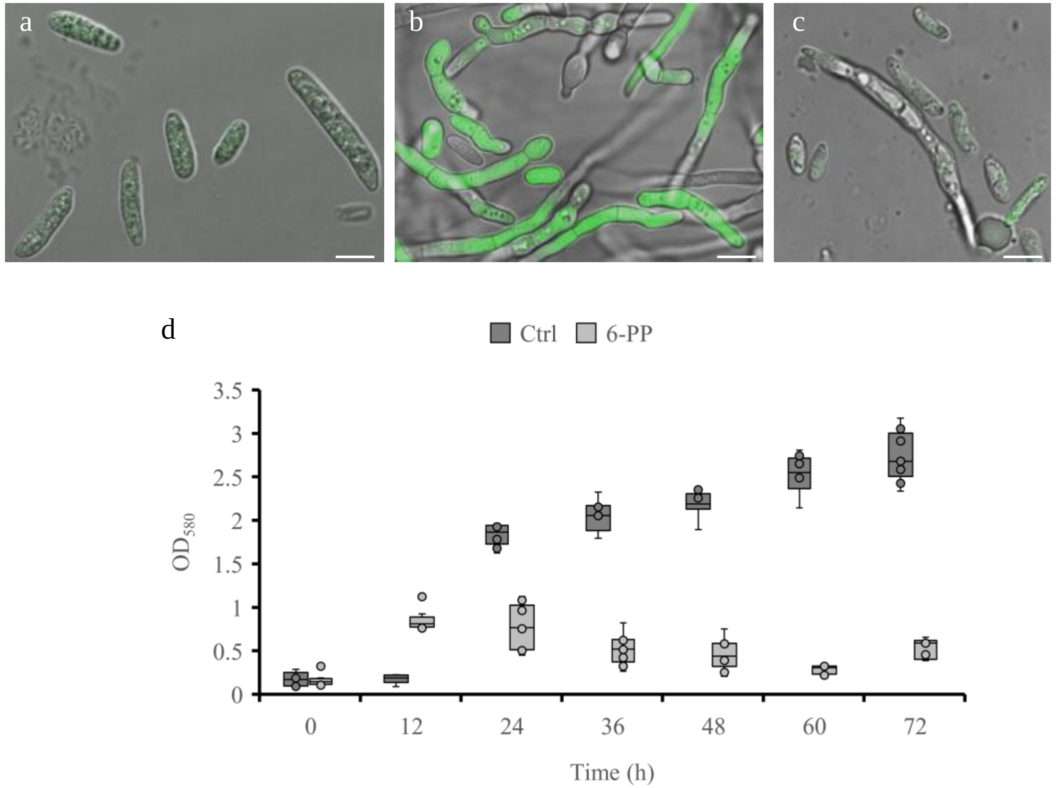

Supplementary Figure 1. Xerucitrinin A and 6-pentyl-α-pyrone isolated from *S. morookaensis* Sm4-1986 show antifungal activity against *Foc* TR4. a, GFP-tagged *Foc*TR4 was cultured in PDB containing 3 mM xerucitrinin A for 36 h, showing faded fluorescence. b, GFP-tagged *Foc*TR4 grown in PDB for 36 h showing fluorescence. c, GFP-tagged *Foc*TR4 was cultured in PDB with 0.96 mM 6-pentyl-α-pyrone for 36 h, showing weakened GFP fluorescence. Scale bars, 5 µm. d, pH values of the PDB solutions with or without 0.96 mM 6-pentyl-α-pyrone. The mean of triplicate and the standard deviation were shown.

Supplementary Figures and Tables

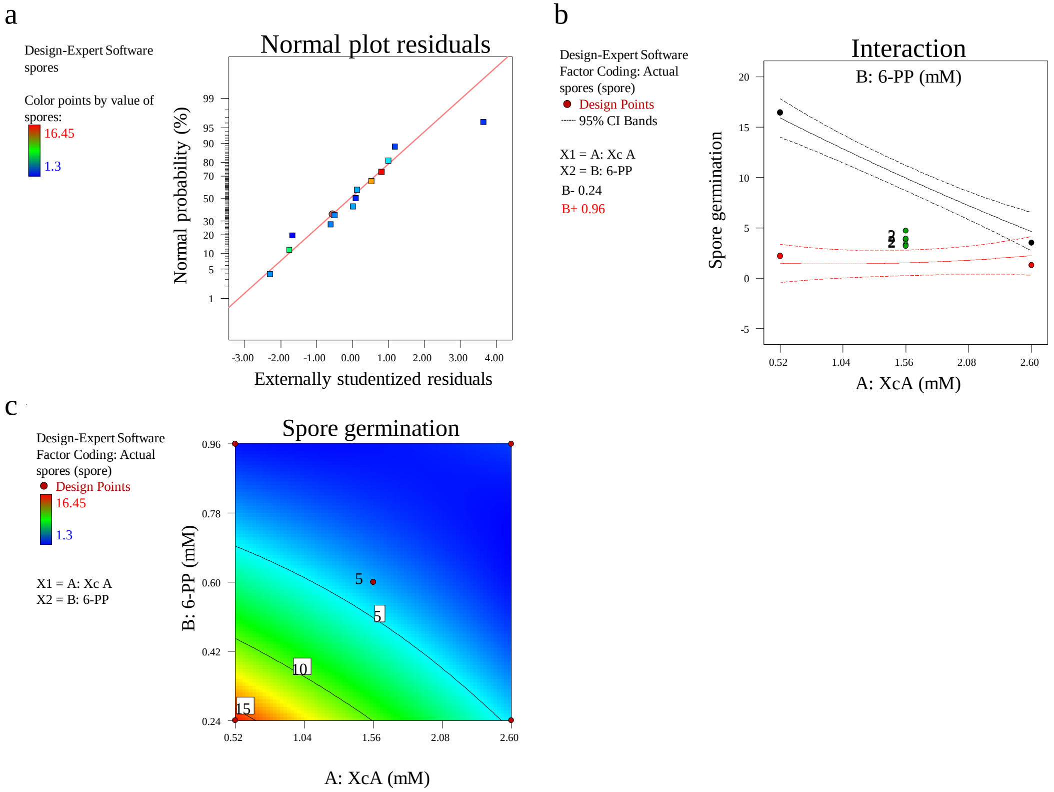

Supplementary Figure 2. Response surface methodology analysis of the mutual enhancement of xerucitrinin A and 6-pentyl-α-pyrone in inhibiting *Foc* TR4 spore germination. a, Normal probability of internally studentized residuals. b, Xerucitrinin A and 6-pentyl-α-pyrone interaction diagram. c, Contour plots for the effects of xerucitrinin A and 6-pentyl-α-pyrone on germination of *Foc* TR4 spores.

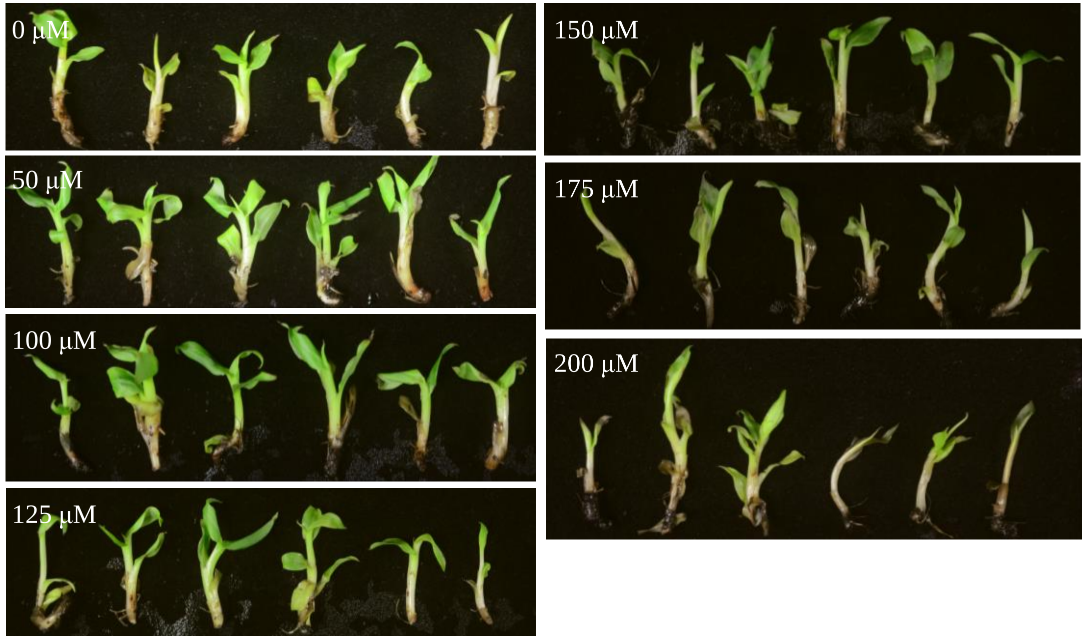

Supplementary Figure 3. Dosage-dependent effects of 6-pentyl-α-pyrone on the growth of banana plantlets. Micro-propagated banana plantlets were grown in 1/2 MS medium without or with different concentrations of 6-PP for 10 days. Low concentrations of 6-pentyl-α-pyrone (50-150 μM) promote growth of banana plantlets, whereas high concentrations of 6-pentyl-α-pyrone (200 μM) show side effects to banana plantlets.

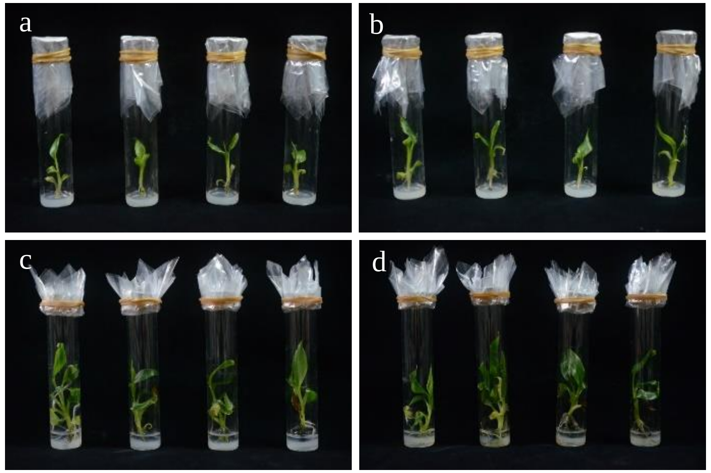

Supplementary Figuer 4. Effect of xerucitrinin A on banana plantlets. a and b, Micro-propaged banana plantlets grown in ½ MS medium without 3 mM xerucitrinin A (a) and with 3 mM xerucitrinin A (b) at 0 day. c and d, Banana plantlets in (a) and (b) grown for 65 days. Equal amount of ddH_2O were added to each tube during the growth period.

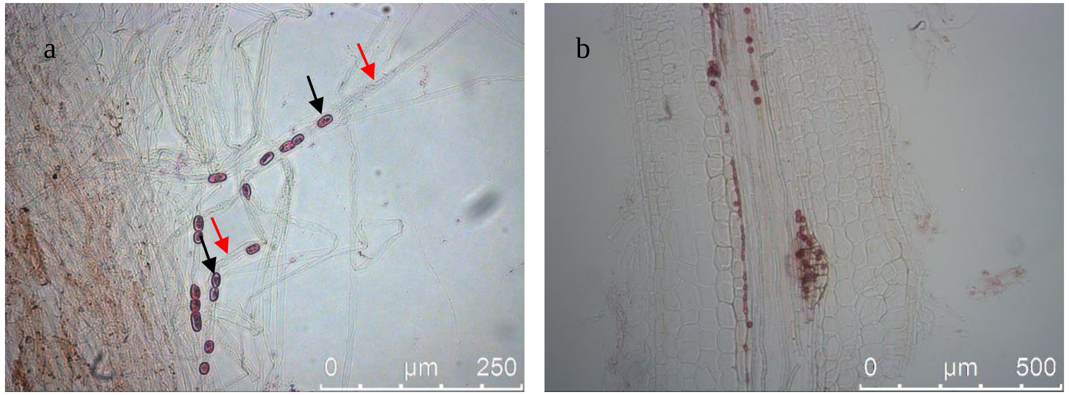

Supplementary Figure 5. Colonization pattern of *P. indica* in banana roots. a, *P. indica* chlamydospores (indicated by the black arrows) were observed in the root hairs (indicated by the red arrows) of banana plants. b, *P. indica* chlamydospores colonize the stele of banana roots and aggregate in the lateral root primordium initiation site.

Supplementary Figures and Tables

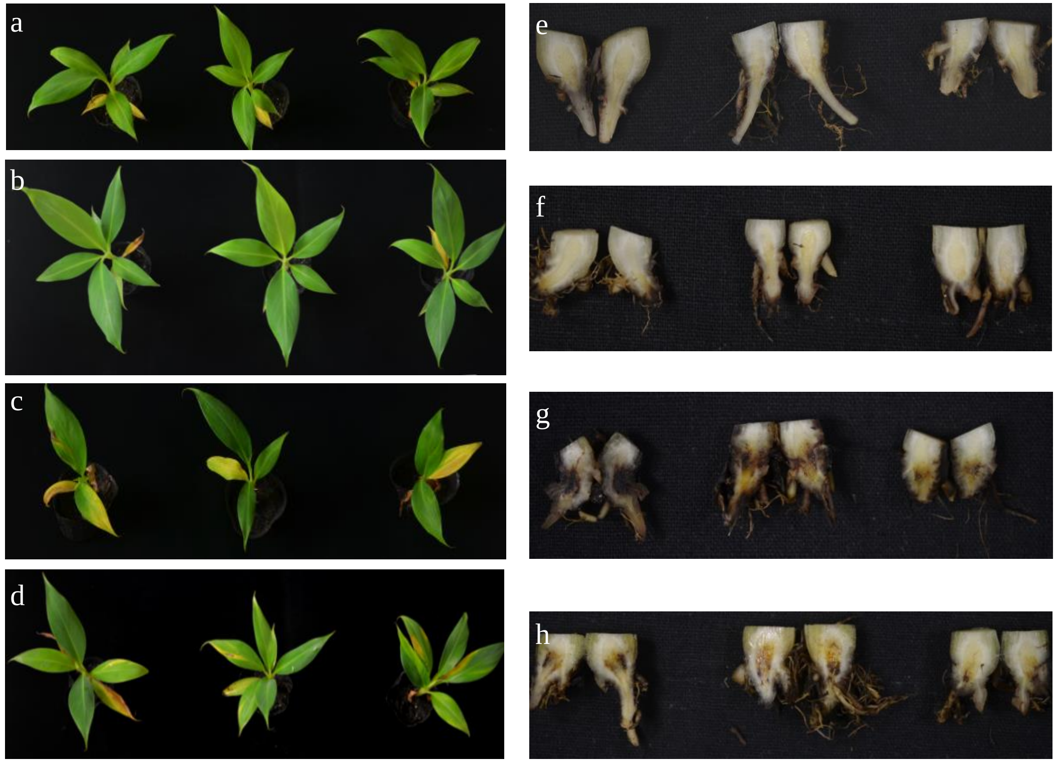

Supplementary Figure 6. *P. indica* promotes banana growth and reduces Fusarium wilt disease symptom. a, Cavendish banana plantlets grown in greenhouse as controls. b, Banana plantlets treated with *P. indica* (1×10^6 chlamydospores/ml) grew better than control plants. c, *Foc* TR4-treated banana plantlets (1×10^6 conidia/ml) showed Fusarium wilt symptom of leaf yellowing. d, Banana plantlets first treated by *P. indica*, and then inoculated by *Foc* TR4 one week later. The leaf yellowing symptom is reduced. e, Clean internal rhizomes of the control plants in (a). f, Clean internal rhizomes of the plants in (b). g, The internal symptom of discoloration in the rhizomes of the plants in (c). h, The internal symptom of less discoloration in the rhizomes of the plants in (d).

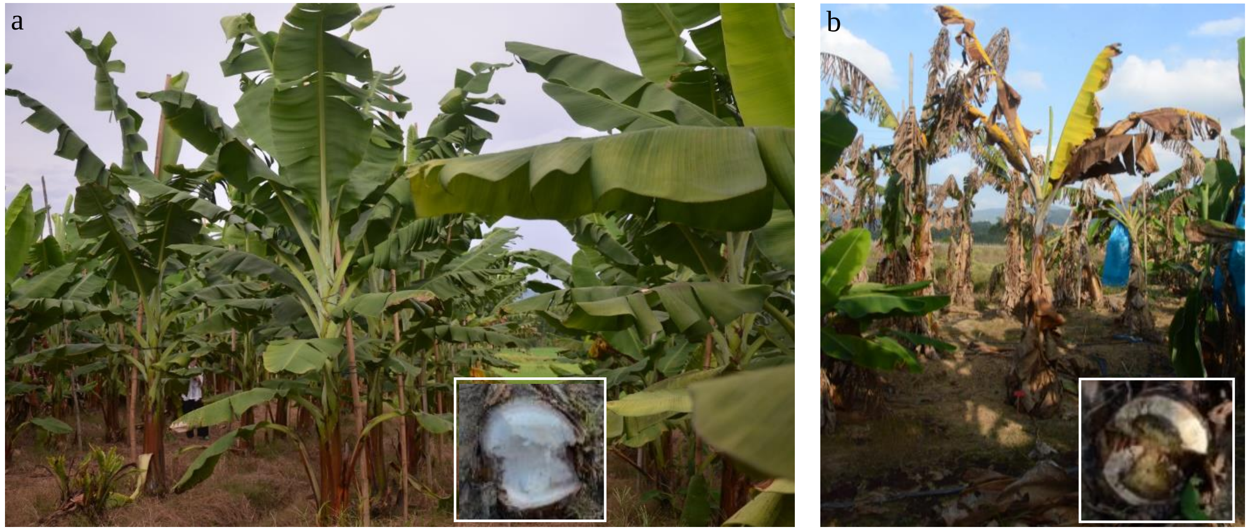

Supplementary Figure 7. Biocontrol of FWB in the field. a, Banana plants that were inoculated by *P. indica* and transplanted in the field treated by *S. morookaensis* strain 4-1986 did not show Fusarium wilt disease. b, Untreated banana plants that were grown in the untreated holes showed Fusariun wilt disease. The white boxed inserts showed the inside of pseudostems.

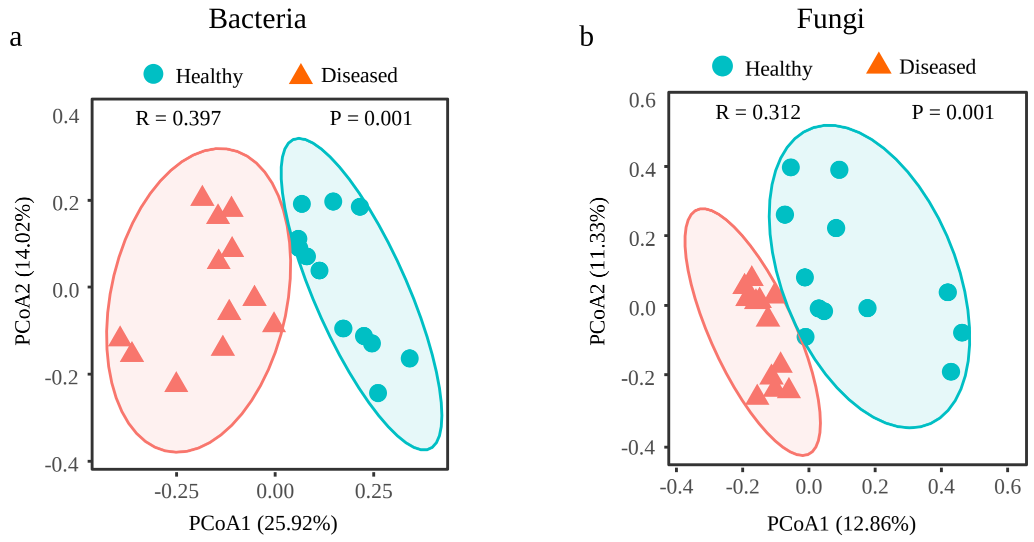

Supplementary Figure 8. PCoA analysis of rhizosphere microbes in the first year of biocontrol. a and b, Principal coordinate analysis (PCoA) of bacterial community (a) and fungal community (b) in the rhizosphere soil between healthy (*n* = 12) and diseased (*n* = 12) plants based on the Bray-Curtis distance, and each symbol represents an individual.

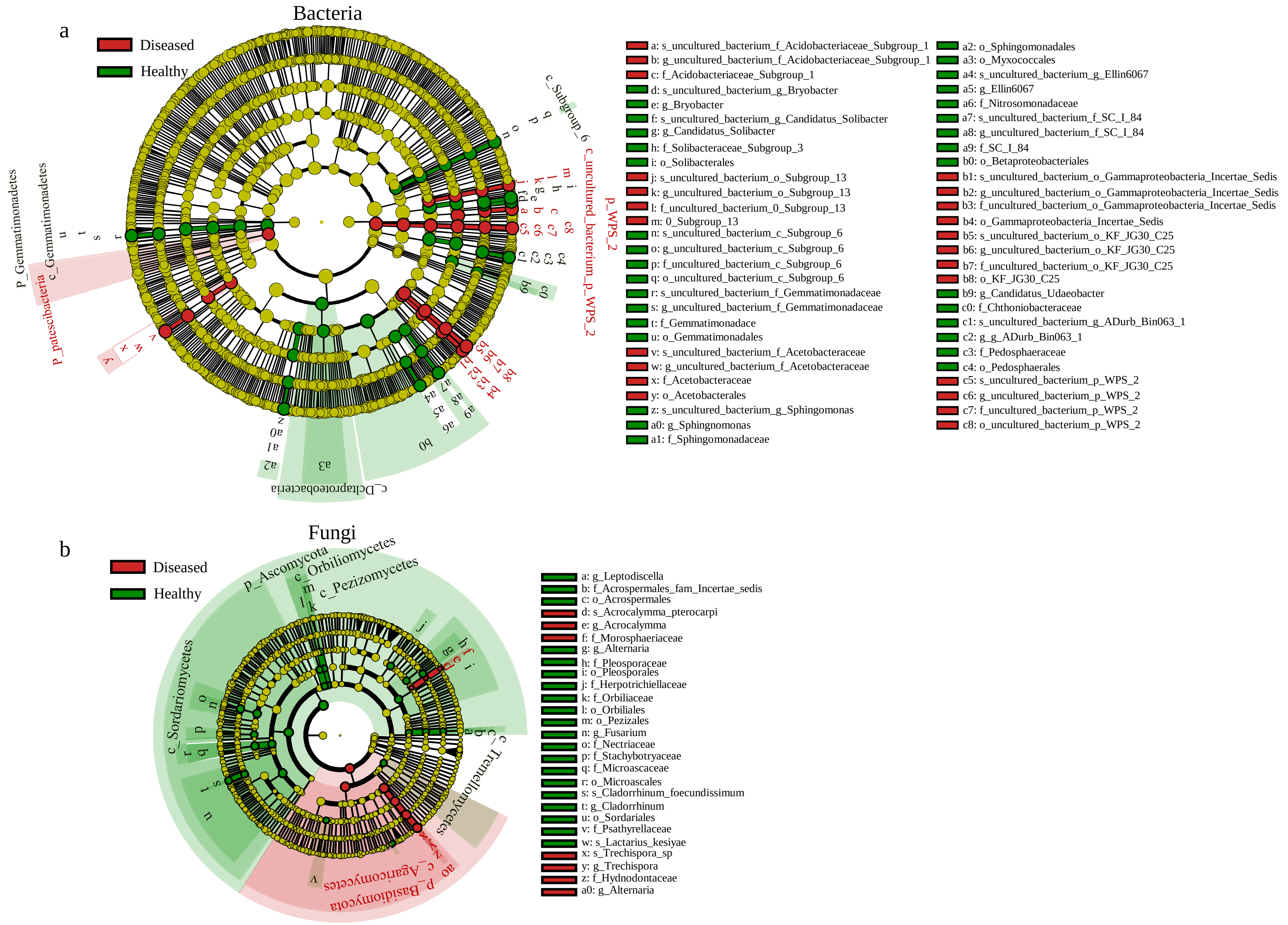

Supplementary Figure 9. Linear discriminant analysis effect size (LEfSe) cladograms showing soil bacterial (a) and fungal (b) abundance (biomarkers) between the healthy and diseased plants in the second year of biocontrol of FWB in the field. The diameter of each circle is proportional to the abundance.

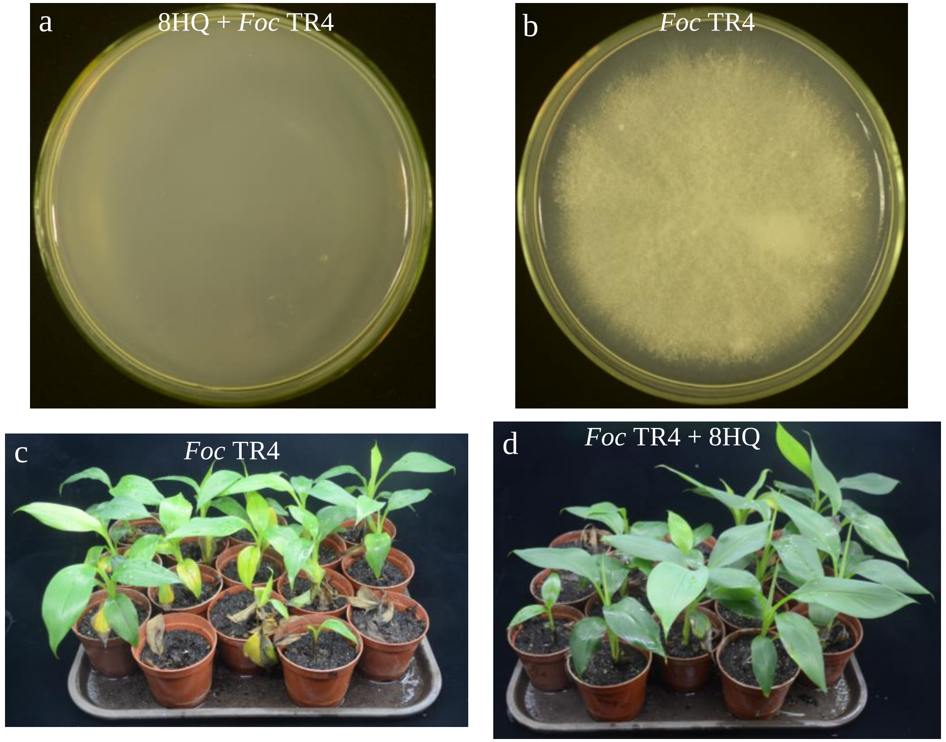

Supplementary Figure 10. The pot trials in greenhouse showing that iron is a crucial factor in control of FWB. a, 200 μM 8HQ (8-Hydroxyquinoline) completely suppressed *Foc* TR4 growth in a PDA plate. b, *Foc* TR4 grew normally in a PDA plate. c, Banana plantlets in pots infected by *Foc* TR4 showed Fusarium wilt disease of leaf yellowing after growing for 75 days. d, Banana plantlets in pots first infected by *Foc* TR4 and then treated by 200 μM 8HQ did not show Fusarium wilt disease after growing for 75 days.

Supplementary Table 1

Table 1. Effects of *P. indica* and *Foc* TR4 treatments on growth of banana plantlets

Character	Ctrl	Pi	*Foc* TR4	Pi + *Foc* TR4
Fresh root weight (g)	2.94±0.36a	4.17±0.15b	1.67±0.12c	3.18±0.28a
Fresh pseudostem weight (g)	5.55±0.38a	8.38±0.12b	3.83±0.16c	5.57±0.02a
Plantlet height (cm)	24.33±0.66a	28.66±0.33b	20.66±0.33c	23.00±0.57d

Notes: Experiment was repeat three times with similar results. Each treatment contained 15 plantlets. the means and standard errors were shown. ANOVA was used to analyze data and LSD comparison was used to detect significance. Different letters behind the numbers in the same row indicate statistical significance at $P < 0.05$.

Supplementary Table 2

Table 2 Comparison of the alpha-diversity indexes of the rhizosphere microbiome from diseased, healthy and bio-treated plants in two years

Soil Sample		Community indexes					
		Bacterial community			Fungal community		
		ACE	Chao1	Shannon	ACE	Chao1	Shannon
Year1	Diseased	1427.53a	1437.24a	4.97a	248.69a	258.18a	3.07a
	Healthy	1476.76a	1505.97a	7.34b	347.46b	369.37b	5.19b
	Bio-treated	1441.34a	1464.92a	7.61b	344.63b	353.95b	5.41b
Year2	Diseased	1430.61a	1463.72a	8.41a	471.61a	424.12a	3.78a
	Healthy	1743.84b	1769.22b	8.96b	585.64ab	489.06ab	5.72b
	Bio-treated	1769.94b	1805.36b	8.93b	614.41b	520.62b	6.33b

Note：ANOVA was used to analyze data and LSD comparison was used to detect significance. Different letters behind the numbers in the same column of the same year indicate statistical significance at $P < 0.05$.

Supplementary Table 3

Table 3 Properties of the rhizosphere soil from diseased, healthy and bio-treated plants in two years

	Soil sample	pH	SM (%)	TN (%)	TP (%)	TF (%)
Year 1	Diseased	5.02±0.014a	0.24±0.003a	0.16±0.006a	0.08±0.002a	0.08±0.001a
	Healthy	5.89±0.015b	0.26±0.011a	0.21±0.005b	0.11±0.006b	0.06±0.002b
	Bio-treated	5.81±0.165b	0.25±0.015a	0.22±0.004b	0.12±0.002b	0.06±0.007b
Year 2	Diseased	5.45±0.047a	0.15±0.005a	0.09±0.005a	0.06±0.001a	0.08±0.003a
	Healthy	5.99±0.087b	0.14±0.012a	0.17±0.008b	0.10±0.009b	0.04±0.005b
	Bio-treated	5.92±0.074b	0.15±0.008a	0.15±0.010b	0.09±0.006b	0.05±0.002b

Note： Experiment was repeat three times with similar results. The means and standard errors were shown. ANOVA was used to analyze data and LSD comparison was used to detect significance. SM, soil moisture; TN, total nitrogen; TP, total phosphorus; TF, total ferrum. Different letters behind the numbers in the same column of the same year indicate statistical significance at $P < 0.05$.
