# Supplementary material for: Spatiotemporal biocontrol and rhizosphere microbiome analysis of Fusarium wilt of banana
Source: Commun Biol. 2023 Jan 11;6:27. doi: 10.1038/s42003-023-04417-w (PMC9834294; doi:10.1038/s42003-023-04417-w)
Supplement: Supplementary file 3 — Description of Additional Supplementary Files [file 42003_2023_4417_MOESM3_ESM.pdf]

Description of Additional Supplementary Files

File name: Supplementary Data 1

Description: Accession numbers for sequence data used in the analysis

File name: Supplementary Data 2

Description: The source data behind the graphs in the paper
