# Supplementary material for: Spatiotemporal biocontrol and rhizosphere microbiome analysis of Fusarium wilt of banana
Source: Commun Biol. 2023 Jan 11;6:27. doi: 10.1038/s42003-023-04417-w (PMC9834294; doi:10.1038/s42003-023-04417-w)
Supplement: Supplementary file 6 — Reporting Summary [file 42003_2023_4417_MOESM6_ESM.pdf]

nature research

Corresponding author(s): Zhihong Tian, Jianxiong Li

Last updated by author(s): Sep 18, 2022

Reporting Summary

Nature Research wishes to improve the reproducibility of the work that we publish. This form provides structure for consistency and transparency in reporting. For further information on Nature Research policies, see our Editorial Policies and the Editorial Policy Checklist.

Statistics

For all statistical analyses, confirm that the following items are present in the figure legend, table legend, main text, or Methods section.

n/a	Confirmed	
☐	☒	The exact sample size (*n*) for each experimental group/condition, given as a discrete number and unit of measurement
☐	☒	A statement on whether measurements were taken from distinct samples or whether the same sample was measured repeatedly
☐	☒	The statistical test(s) used AND whether they are one- or two-sided<br>*Only common tests should be described solely by name; describe more complex techniques in the Methods section.*
☒	☐	A description of all covariates tested
☐	☒	A description of any assumptions or corrections, such as tests of normality and adjustment for multiple comparisons
☐	☒	A full description of the statistical parameters including central tendency (e.g. means) or other basic estimates (e.g. regression coefficient) AND variation (e.g. standard deviation) or associated estimates of uncertainty (e.g. confidence intervals)
☐	☒	For null hypothesis testing, the test statistic (e.g. *F*, *t*, *r*) with confidence intervals, effect sizes, degrees of freedom and *P* value noted<br>*Give P values as exact values whenever suitable.*
☒	☐	For Bayesian analysis, information on the choice of priors and Markov chain Monte Carlo settings
☒	☐	For hierarchical and complex designs, identification of the appropriate level for tests and full reporting of outcomes
☒	☐	Estimates of effect sizes (e.g. Cohen's *d*, Pearson's *r*), indicating how they were calculated

Our web collection on statistics for biologists contains articles on many of the points above.

Software and code

Policy information about availability of computer code

Data collection	No software were used for data collection.
Data analysis	Analysis were performed using SPSS Statistics version 20.0 and R 3.2.0 software.

For manuscripts utilizing custom algorithms or software that are central to the research but not yet described in published literature, software must be made available to editors and reviewers. We strongly encourage code deposition in a community repository (e.g. GitHub). See the Nature Research guidelines for submitting code & software for further information.

Data

Policy information about availability of data

All manuscripts must include a data availability statement. This statement should provide the following information, where applicable:

- Accession codes, unique identifiers, or web links for publicly available datasets
- A list of figures that have associated raw data
- A description of any restrictions on data availability

If published, all data will be made available at the Dryad Digital Repository. All sequence data that of this study have been deposited in NCBI Sequence Read Archive database and all the accession codes are provided in online methods.

Field-specific reporting

Please select the one below that is the best fit for your research. If you are not sure, read the appropriate sections before making your selection.

☐ Life sciences ☐ Behavioural & social sciences ☒ Ecological, evolutionary & environmental sciences

For a reference copy of the document with all sections, see nature.com/documents/nr-reporting-summary-flat.pdf

Ecological, evolutionary & environmental sciences study design

All studies must disclose on these points even when the disclosure is negative.

Study description	Here we conducted a series of experiments that, at different levels and scales, showed an efficient method and the key factors to controlling Fusarium wilt of banana, and the richness and changes of the rhizosphere microbiome in the process of biocontrol of Fusarium wilt of banana. All experimental treatments in the laboratory and greenhouse were replicated at least three times and the trial in the field was repeated in two years. Each experiment is described in more detail in the methods section of the manuscript.
Research sample	We used the soil samples collected from the roots of banana plants in the field at the time that the Fusarium wilt disease symptoms were easily distinguished after the the application of biocontrol agents. The samples were further used to analyze rhizosphere microbiome and soil property.
Sampling strategy	Sampling in the field was based on clear phenotypes of banana plants. Both external and internal symptoms were investigated to confirm that the banana plants either are Fusarium wilt diseased or healthy. Samples were taken from the representative plants of the diseased and healthy groups.
Data collection	All data were collected by ZYZ and JXL.
Timing and spatial scale	All data were collected in the years of 2019 and 2020 in China and the field experiments were conducted in a land of 0.8 hectares.
Data exclusions	No data excluded.
Reproducibility	Experiments in laboratory and greenhouse were replicated to ensure reproducibility of the results. Field experiments were carried out in two years with consistency in biocontrol efficacy.
Randomization	Field experiments were contrast design. Fifteen plants in a line were treated as a block, and the treatment and control blocks were arranged alternately in field.
Blinding	Blinding was not relevant to our study. Banana plants needed to be clearly distinguished between Fusarium wilt diseased and healthy, and the representatives of each group were chosen for analyses.

Did the study involve field work? ☒ Yes ☐ No

Field work, collection and transport

Field conditions	The experiments were conducted in the whole year of 2019 and 2020 in a land that had been cultivated banana for more than 10 years and recently abandoned by the farmer due to the severe infection of Foc TR4. The soil type is acidified red soil in Southern China. Standard irrigation and fertilization practices were applied to banana plants, and other managements were followed the normal farm operations in banana orchard.
Location	Longmen County (23°72'77"N, 114°25'49"), Huizhou City, Guangdong Province, China
Access & import/export	Access and import/export our samples were permitted by the landowner.
Disturbance	No disturbance caused by the study.

Reporting for specific materials, systems and methods

We require information from authors about some types of materials, experimental systems and methods used in many studies. Here, indicate whether each material, system or method listed is relevant to your study. If you are not sure if a list item applies to your research, read the appropriate section before selecting a response.

Materials & experimental systems

n/a	Involved in the study
☒	☐ Antibodies
☒	☐ Eukaryotic cell lines
☒	☐ Palaeontology and archaeology
☒	☐ Animals and other organisms
☒	☐ Human research participants
☒	☐ Clinical data
☒	☐ Dual use research of concern

Methods

n/a	Involved in the study
☒	☐ ChIP-seq
☒	☐ Flow cytometry
☒	☐ MRI-based neuroimaging
